# Diversity and Function of Somatostatin-Expressing Interneurons in the Cerebral Cortex

**DOI:** 10.3390/ijms20122952

**Published:** 2019-06-17

**Authors:** Therese Riedemann

**Affiliations:** Ludwig-Maximilians-University, Biomedical Center, Physiological Genomics, Großhaderner Str. 9, 82152 Planegg-Martinsried, Germany; therese.riedemann@med.uni-muenchen.de; Tel.: +49-89-2180-75211

**Keywords:** interneuron classification, GABA, Somatostatin, Martinotti cell, mood disorders

## Abstract

Inhibitory interneurons make up around 10–20% of the total neuron population in the cerebral cortex. A hallmark of inhibitory interneurons is their remarkable diversity in terms of morphology, synaptic connectivity, electrophysiological and neurochemical properties. It is generally understood that there are three distinct and non-overlapping interneuron classes in the mouse neocortex, namely, parvalbumin-expressing, 5-HT_3A_ receptor-expressing and somatostatin-expressing interneuron classes. Each class is, in turn, composed of a multitude of subclasses, resulting in a growing number of interneuron classes and subclasses. In this review, I will focus on the diversity of somatostatin-expressing interneurons (SOM^+^ INs) in the cerebral cortex and elucidate their function in cortical circuits. I will then discuss pathological consequences of a malfunctioning of SOM^+^ INs in neurological disorders such as major depressive disorder, and present future avenues in SOM research and brain pathologies.

## 1. Introduction

### Brief Overview on the Diversity of GABAergic Interneurons in the Brain

Two main neuron populations are found within the rodent and human cerebral cortex: excitatory projection neurons and inhibitory interneurons. Their relative numbers differ from brain region to brain region but generally, excitatory projection neurons make up around 70–80% of the overall neuron population, whereas inhibitory interneurons constitute the remaining 20–30%. All exceptions aside, most excitatory projection neurons use the neurotransmitter glutamate and the larger portion of excitatory projection neurons are pyramidal neurons. The great majority of inhibitory interneurons in turn expresses γ-amino-butyric acid (GABA). In contrast to pyramidal neurons that represent a rather homogenous group of cells, GABAergic interneurons are characterized by a stand-alone diversity of cellular properties ranging from the expression of different neurochemical marker proteins and a great variety of morphological phenotypes to highly varying electrophysiological properties [1,2,3]. A common feature that is shared by the vast majority of inhibitory interneurons is the fact that their axonal projections do not leave their respective brain area, hence their original name ’short axon cells’ put forward by Ramon y Cajal. Inhibitory interneurons whose axons project across their home brain area are called inhibitory long-range projection neurons [4] and, strictly speaking, long-range GABAergic projecting neurons are not considered interneurons, going by their original name. Some neurological and/or neuropsychiatric diseases are correlated with a specific loss of a certain type of interneurons [5,6,7,8], strongly suggesting that certain types of neurons play distinct roles within a given neocortical circuit. In order to understand why a certain type of cell seems more susceptible to a neuropathological condition compared to others, it is necessary to unravel their specific cellular and neural circuit properties. The function of a certain cell type within a given neural circuit in a given brain area needs to be studied. Therefore, a thorough characterization of all properties that make up a certain type of interneuron must be the basis of all classification schemes. However, a problem that every classification attempt encounters is that classifications are only based on the properties that are visible to the respective observer, possibly neglecting other important features that the observer is blind to (because analysis of certain properties was not included in the experimental design). Most experiments are restricted to the analysis of a limited number of features, hence the basis for most classification schemes is based on neurochemical and/or morphological and/or electrophysiological and/or genomic properties of a given cell aggravating a common nomenclature. In addition, a possible function of a certain class of cells can often not be derived from a descriptive classification study. Principally, each cortical neuron can be defined by its cell-type specific properties ranging from its location within the cortex, its neurochemical composition, its synaptic input and output, its endowment with certain types of ion channels to its action potential discharge behavior. But given that researchers will not be able to analyze every cell feature of a given cell type it is necessary to understand and determine which particular properties are sufficient to assign each cell an overarching class, either within a given brain area or even across different brain areas or even across different species [9]. In addition, the question remains whether cellular differences found within the population of a given interneuron class truly reflect different ’species’ or are the result of a certain degree of within-group variability. Despite these challenges, the classification of GABAergic interneurons has made tremendous progress. Classification studies that were mostly based on neuron morphology revealed the existence of less than 10 interneuron classes, the most prominent being (1) basket cells, (2) Martinotti cells, (3) neurogliaform cells, (4) chandelier cells, (5) Cajal-Retzius cells, (6) double-bouquet cells (also named horse-tail cells) and (7) long-range GABAergic projecting cells. Electrophysiological classification studies were mostly based on a cell’s passive membrane properties, its single spike kinetics and on its action potential discharge behavior and six electrophysiological classes of interneurons were proposed: (1) fast-spiking cells, (2) non-adapting non-fast-spiking, (3) adapting cells, (4) accelerating cells, (5) irregular-spiking cells and (6) intrinsically bursting cells [2,3,10], each class consisting a several subtypes [11]. However, given that firing patterns are dynamic and that there is a fluid transition between different firing patterns, a categorization based on firing patterns is challenging [12].

In recent years, increased efforts were undertaken to define transcriptomic cell types via single-cell RNA sequencing and up to date eight types of GABAergic cells were found, six of these types being composed of multiple subtypes, adding up to more than 15 subtypes of GABAergic interneurons [13,14,15]. In many cases, class affiliation complied with the embryonic origin of a given cell type [14,16].

**Figure 1:**
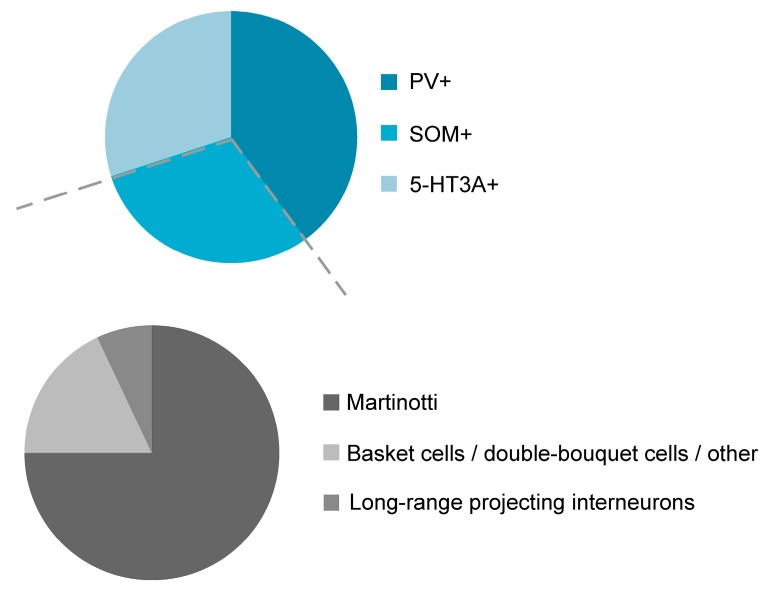
GABAergic interneurons are divided into three distinct and non-overlapping neurochemical classes: Parvalbumin-containing (PV^+^), somatostatin-expressing (SOM^+^), and 5-HT_3A_-receptor-containing (5-HT_3A_ receptor^+^) interneurons (upper panel). The class of SOM^+^ interneurons in turn is divided into Martinotti cells and non-Martinotti cells that can be subdivided into long-range projecting interneurons and, among others, basket cells and double-bouquet cells (lower panel).

The classification of interneurons according to their neurochemical properties is becoming increasingly popular, because it allows the genetic manipulation in a subset of cells that is targeted by their cell-type-specific genes and classification studies based on neurochemical properties suggest three non-overlapping interneuron classes in the mouse neocortex, namely parvalbumin-expressing (PV^+^ INs), somatostatin-expressing (SOM^+^ INs) and 5-HT_3A_-receptor-expressing interneurons (5HT_3A_-receptor^+^ INs; [17,18,19]; Figure 1), each class consisting of several subgroups [20,21,22]. In addition, the group of 5HT_3A_ receptor-expressing interneurons in turn can be divided into vasoactive intestinal peptide-expressing (VIP) and VIP-lacking cells [17,18]. Classification studies that are based on the combination of morphological, electrophysiological and/or neurochemical properties will accordingly yield an even higher number of different cell types. In the hippocampus, at least 10 different GABAergic cell types were proposed [9] and it is likely that the same number of interneuron types exists in the neocortex [11,23]. This review will focus on the diversity and function of SOM^+^ INs within the neocortex.

## 2. Development of SOM^+^ Interneurons

The great majority of inhibitory interneurons are generated in the ganglionic eminences and in the preoptic area [24,25,26,27,28,29,30]. In contrast to pyramidal neurons that reach their target area by radial migration, cortical inhibitory interneurons take a tangential path to arrive at their destined location within a given brain area. In the mouse, the generation of cortical interneurons occurs in two waves dependent on their place of birth within the ganglionic eminences: the majority of cortical interneurons expressing either parvalbumin (PV) or somatostatin (SOM) arises from the medial ganglionic eminences (MGE) and is born between embryonic days E10.5 and E16.5 [31,32]. MGE-derived cortical interneurons are born in a sequential fashion and with a topographical bias where the generation of prospective SOM-expressing interneurons preferentially occurs in the dorsal division of the MGE and peaks at E14.5 whereas that of PV-expressing interneurons takes place in the ventral division of the MGE and reaches its maximum only at E15.5 [32,33,34,35,36]. The caudal ganglionic eminence (CGE) in turn gives mainly rise to VIP- and/or 5-HT_3A_ receptor-expressing interneurons and to some degree also to SOM- and/or neuropeptide Y- (NPY^+^) expressing interneurons [37,38,39]. CGE-derived interneurons are mostly born after E15.5 and represent the group of late-born cortical interneurons. Cortical interneurons that are derived from the preoptic area in turn develop into a wide variety of cortical interneurons with no obvious preference for a certain subtype of cells [30]. MGE-derived interneurons represent around 60% of adult cortical inhibitory interneurons, while CGE-derived interneurons give rise to around 30% of all cortical GABAergic interneurons. The POA in turn contributes around 10% of interneurons to the adult cortical interneuron population [30]. Several genes that are widely expressed within the ganglionic eminences control the generation and/or maturation of cortical interneurons. The transcription factors Ascl-1 and Dlx-1/-2 for example are strongly expressed in the ventricular and subventricular zones of the ganglionic eminences and it appears that loss of either transcription factor leads to a pronounced loss of cortical interneurons [40,41,42]. In addition, a fate-specification for MGE-derived cortical interneurons to become either PV+ or SOM^+^ requires a distinct expression of transcription factors in either cell. It is suggested that the transcription factor CoupTF2 in the MGE and POA establishes SOM^+^ IN fate by promoting Sox6 expression in MGE progenitors [43,44] and conditional deletion of CoupTF2 increases the numbers of PV^+^ interneurons [44]. In addition, it appears that only the continued expression of Sox6 during migration of SOM^+^ INs maintains a SOM^+^ IN identity [45]. However, to complicate matters, clonal analysis revealed that at least some SOM^+^ and PV^+^ interneurons are born from the same progenitor clone [46]. In summary, it is likely that a distinct combination of transcription factors is necessary to induce SOM^+^ fate but it is currently unclear how these transcription factors promote the induction of SOM versus PV fate [47]. The specific properties of any newborn cell will be refined once it has reached its final destination within the cerebral cortex and has established synaptic connections within its given neural circuit.

## 3. Distribution and Morphological Variety of SOM^+^ Interneurons

### 3.1. Distribution of SOM^+^ INs

As can be seen from Figure 1, PV^+^ INs represent the largest interneuron population in the neocortex and make up around 40–50% of all GABAergic interneurons [18,20,21,48]. PV^+^ INs are densely located in cortical layer 4, but are also found in higher numbers in cortical layer 2/3 and layer 5 [18]. SOM-expressing interneurons (SOM^+^ INs) in turn, constitute around 30% of all GABAergic interneurons. The remaining fraction of interneurons are 5-HT_3A_ receptor- and/or VIP-expressing interneurons. It appears that these three neurochemical classes are virtually non-overlapping with exceptions reported in the mouse subiculum [49], the entorhinal cortex [50], the endopiriform nucleus [51], the olfactory bulb [52], the amygdala [53] and the hippocampus [54]. In layers 2 and 3 of the cingulate cortex [22] but likely also in other cortical areas [20,55], SOM-expressing interneurons represent around 2–3% of the overall neuron population. With the exception of layer 1, SOM^+^ INs are present in all cortical layers [18,20,56,57,58,59]. The distribution of SOM^+^ cortical interneurons was mostly established in the mouse visual and somatosensory cortex but is likely to hold true for other cortex areas as well. Indeed, the distribution of SOM^+^ INs in the agranular rat frontal cortex follows a similar pattern [21]. With regard to morphology, SOM^+^ INs display a variety of phenotypes, the most prominent ones being those described in the following sections. 

### 3.2. Morphological Variety of SOM^+^ INs

**Figure 2:**
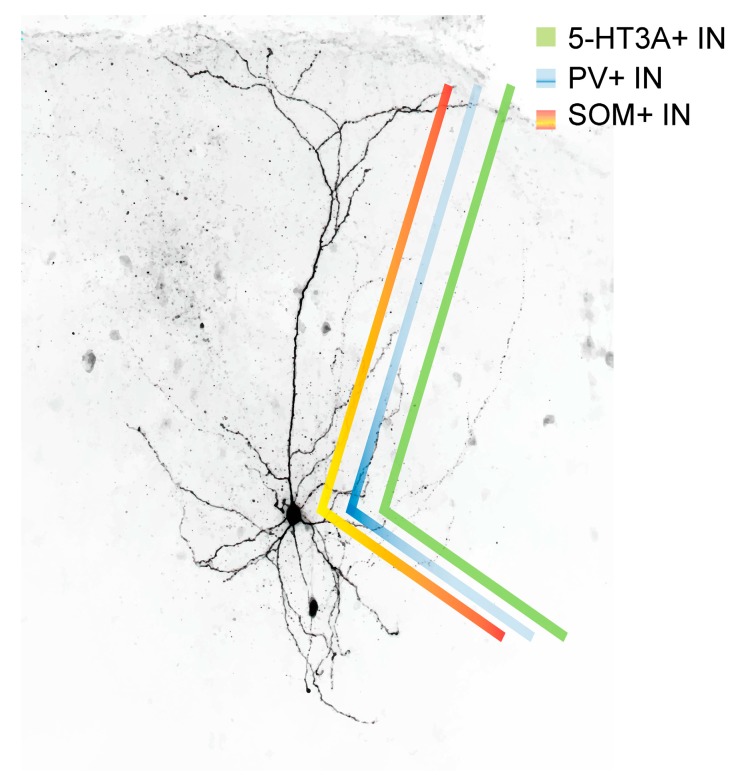
Postsynaptic target density of PV^+^ (blue), SOM^+^ (red) and 5-HT_3A_ receptor^+^ interneurons (green). While PV^+^ interneurons mainly target the proximal dendrites and soma of a given pyramidal cell, 5-HT_3A_ receptor-containing interneurons target, depending of the 5-HT_3A_ receptor^+^ cell subtype, rather the distal and/or proximal dendrite and/or the soma. SOM^+^ INs in turn target primarily the distal dendrites of a given pyramidal cell.

Morphologically, the class of SOM-expressing interneurons can be divided into two broad subclasses: Martinotti and non-Martinotti cells (Figure 1; [59]). SOM^+^ INs of the non-Martinotti type comprise, among others, basket cells, double-bouquet cells and long-range GABAergic projection cells [4,12,57,60,61,62] (Figure 2). The axonal projections of basket cells remain largely within a given cortical layer where they can ramify extensively and target the majority of neighboring pyramidal neurons within a 100 µm radius [63]. Their name is derived from their basket-like presynaptic envelopes that they seem to wrap around the cell bodies of postsynaptic pyramidal neurons [64]. In terms of their morphology and size, basket cells are divided into large basket cells, small basket cells and nest basket cells, the specific properties of each of these subclasses not being discussed here. Commonly, basket cells are associated with parvalbumin expression, but SOM immunoreactivity has been shown in a small fraction of any basket cell type [65]. Altogether, basket cells constitute around one half of all supragranular GABAergic cortical interneurons [11]. Double-bouquet cells are mainly found in cortical layers 2–5 with a bias towards supragranular cortical layers. Their axons form tightly intertwined bundles of descending and sometimes ascending collaterals that can cross several layers [66]; as such, they seem to play a role in interlaminar and intracolumnal inhibition. Rather than with somatostatin immunoreactivity, double-bouquet cells have traditionally been associated with calbindin expression [67]. Nonetheless, SOM expression has been described in a fraction of double-bouquet cells [65]. Given their morphological appearance, double-bouquet cells are regarded as subgroup of VIP-expressing or CCK-expressing interneurons [68].

Another subpopulation of cortical non-Martinotti SOM^+^ INs represent the so-called long-range GABAergic neurons whose axons can project to remote brain areas. They are mainly found in layer 6 and in the white matter and seem to mostly coexpress the neuronal nitric oxide (NO) synthase [61,69]. Their function is well established in the septo-hippocampal circuit and they are thought to generate rhythmic oscillations in the hippocampus [70,71,72,73]. GABAergic long-range projection neurons in the white matter receive excitatory cortical inputs but seem to lack thalamic inputs [4,74]. However, their role in neocortical circuits remains largely unknown [61]. 

Although precise numbers are missing, Martinotti cells represent the larger fraction of all SOM+ INs, ranging from around 55–100% [12,57,62,75] and around 15% of all GABAergic interneurons are Martinotti cells [65]. However, it should be noted that the relative ratios of Martinotti to non-Martinotti cells in the mouse somatosensory cortex vary within a given cortical layer. While the fraction of Martinotti cells is well beyond 50% in layer 2/3 it is virtually non-existent in layer 4 [57,76] and the great majority of SOM^+^ INs in layer 4 are interneurons with a basket-cell like morphology [57,76]. In contrast to the mouse somatosensory cortex, the rat somatosensory cortex contains a considerable fraction of Martinotti cells in layer 4 [11], probably pointing to species-dependent differences. Martinotti cells usually have spindle or ovoid shaped somata [75] with diverse somatodendritic morphologies [12,77]. The majority of Martinotti cells is of the tufted or multipolar type [12,78]. They are characterized by dense axonal ramifications in their home layer and long translaminar ascending axon collaterals directed towards layer 1 where they spread horizontally up to 2000 µm [4,12,75,78]. Performing an intersect analysis from camera lucida drawings, it could be shown that the axons of neocortical SOM^+^ INs constitute at least three quarters (76%) of GABAergic axons in layer 1 [79]. Given their dense axonal morphology, Martinotti cells can modulate the activity of a large number of pyramidal neurons that also represent their preferential postsynaptic targets. In contrast to other interneurons (chandelier cells, neurogliaform cells), dendrites of Martinotti cells are spinous. 

## 4. Neurochemical Variety of SOM^+^ Interneurons

Many classification studies rely on neurochemical markers to identify a given neuronal subtype. The great majority of SOM^+^ neurons express GABA, but SOM immunoreactivity is not necessarily accompanied by GABA expression [48,50,53]. However, in the neocortex SOM has evolved as a reliable marker for a subclass of GABAergic interneurons. Ideally, a correlation between the expression of a certain neurochemical marker protein and function within a given brain circuit is desired. A variety of different marker proteins are coexpressed with somatostatin. Depending on the animal species and brain area, SOM^+^ INs express the calcium-binding proteins calretinin and/or calbindin and/or parvalbumin: for example, SOM colocalization with calretinin was reported in the mouse but not in the rat neocortex [20,21] and calretinin expression in SOM^+^ INs is higher the mouse cingulate compared to the mouse somatosensory or visual cortex or the mouse hippocampus [18,22,56,58,80]. In addition, a considerable fraction of mouse and rat neocortical SOM^+^ INs expresses calbindin [22,57,81]. Furthermore, the neuropeptide NPY and the neuronal nitric oxide synthase can be coexpressed to varying degrees in SOM^+^ interneurons. While colocalization of NPY in SOM^+^ INs was reported in around one third of SOM^+^ INs in the cingulate cortex [22], less than 10% of SOM^+^ INs coexpress NPY in the somatosensory cortex [57]. In addition, preprodynorphin was proposed as a marker to reliably detect a subpopulation of SOM^+^ INs in the cerebral cortex, among which, but not exclusively, Martinotti cells [82]. Importantly, the expression of multiple neurochemical markers in SOM^+^ INs seems to be the rule rather than the exception [20,21,22] adding further complexity to the diversity of SOM^+^ INs in the neocortex.

Lastly, a fraction of SOM^+^ INs expresses neuronal nitric oxide synthase (nNOS), sometimes in coexpression with the receptor for substance P (SPR, [21,48,83]). While only a small fraction of superficial SOM^+^ INs expresses nNOS, their number is higher in the infragranular cortical layers [21,83]. It appears that SOM^+^ INs coexpressing nNOS as well as substance P receptor specifically represent long-range GABAergic projecting neurons [4].

Given the fact that many different neurochemical markers can be expressed in SOM^+^ INs and that a single SOM^+^ IN can be endowed with different combinations of neurochemical markers, the population of SOM^+^ INs is composed of a large number of neurochemical subtypes. The fact that the population of SOM^+^ INs is neurochemically very diverse and contains numerous subtypes is also reflected by single-cell RNA sequencing studies showing that the class of SOM^+^ INs is composed of 10 distinct types (some of the types consisting of multiple subtypes), possibly reflecting distinct neurochemical types of SOM^+^ INs [13,14]. In summary, it remains to be tested whether the defined types of SOM^+^ INs represent different cell types (reflecting a Linnéan family) with distinct properties within a given brain area or whether they are all part of a more general type, i.e., a Linnéan species, that is split into subtypes largely because of environmental adaptations (e.g., brain area, cortical layer, activity within a brain area, etc.). Other hypotheses with regard to classification of interneurons represent the idea that each cell belongs to a class of its own [84] or that the idea of cell classes should be given up in favor of the assumption of a continuum of cells [85] [neocortex]; [86] [hippocampus]. To date it is not quite clear, whether each neurochemical or molecular subtype also serves a different function within a given neuronal circuit. For a general overview of the function and pharmacological relevance of neurochemical markers in the mammalian brain the interested reader is referred to a recent review by Rees et al. [87]. 

## 5. Electrophysiological Properties of SOM^+^ Interneurons

The basis of most electrophysiological classification studies is the analysis of the passive and active membrane properties, analysis of single spike kinetics as well as determination of the action potential discharge pattern. Despite a high degree of morphological, neurochemical and molecular variability within the SOM^+^ IN population, SOM-expressing interneurons exhibit distinct electrophysiological properties that clearly distinguish them from pyramidal neurons, from fast-spiking interneurons or from VIP-containing cells for example [12,78,88,89,90,91,92,93]. In sum, SOM^+^ INs have a more depolarized membrane potential, a higher input resistance, and a shorter membrane time constant compared to fast-spiking cells, exhibit a higher degree of rectification upon injection of a hyperpolarizing current pulse and show spike kinetics that are slower compared to those of fast-spiking interneurons [78,83,91]. Thus, the entity of SOM^+^ INs separates well from other classes of GABAergic interneurons; however, a closer look within the class of SOM^+^ INs reveals the existence of multiple electrophysiological subclasses. 

### Firing Properties of SOM^+^ INs

The fact that SOM^+^ INs constitute a heterogeneous group of cells is also reflected by their variable discharge patterns. The firing properties of a neuron are commonly investigated by injecting multiple rectangular suprathreshold current pulses into a cell and, among others, analyzing the initial frequency, i.e., the reciprocal value of the first interspike interval, the steady state frequency, the adaptation index, i.e., the ratio of both frequencies (initial and steady-state frequency) and, in most cases, by qualitatively judging the discharge pattern. It should be noted, however, that the same cell can adopt different discharge behaviors depending on stimulus strength and stimulus duration (unpublished observations; [94]). Given the fact that quantitative means to analyze discharge patterns in neurons are challenging (not to say they are impossible, see [95]) and that a qualitative analysis allows room for subjective criteria, cell classification studies that are solely based on discharge properties might not suffice for a full characterization of cell types. In addition, because a clear correlation between a cell’s discharge pattern and its neurochemistry or morphology is missing (at least for SOM^+^ INs), classification studies should try to combine at least two if not all three parameters.

According to their predominant action potential discharge pattern and their ability to fire low-threshold calcium spikes in prefrontal infragranular cortical layers, SOM^+^ INs were initially named low-threshold spiking interneurons (LTS, [75,89,96]). While the majority of infragranular SOM^+^ INs are LTS cells, only around 50% of LTS cells express SOM indicating that a low-threshold calcium spike is not a unique feature of SOM^+^ INs [78]. Supragranular SOM^+^ INs in turn rarely exhibit a low-threshold calcium spike [12,78], rather, the majority of them exhibits a continuous, i.e., regular discharge pattern with frequency adaptation upon injection of a rectangular depolarizing current pulse [12,62,75,78]. Regularly discharging interneurons showing frequency adaptation are called regular-spiking non-pyramidal cells (RSNP). In addition, a small percentage of SOM^+^ INs respond to a depolarizing current pulse with almost no frequency adaptation analogous to a fast-spiking interneuron or exhibit an irregular or stuttering firing pattern [12,75,89,93]. Given the facts that the action potential discharge patterns seem to be very variable in SOM^+^ INs, and that a clear correlation between neuropeptide expression and firing properties is missing, the shape of the spike afterhyperpolarization was proposed a better classifier for SOM^+^ INs. In contrast to fast-spiking interneurons, SOM^+^ INs typically possess a dual component AHP with an early and a late peak [12,93,97]. Initial studies often lacked immunochemical confirmation of a certain cell type and cells were mostly selected based on their somatodendritic appearance. Therefore, early electrophysiological recordings entailed a certain degree of uncertainty of whether recordings were performed from the same subtype of non-pyramidal neuron or not. In addition, given the fact that SOM^+^ INs represent less than 5% of the overall neuron population [22,75] and that the somata of SOM^+^ INs can come in a variety of shapes, chances of patching specifically from a SOM^+^ IN are not very high using infrared microscopy. Therefore, many electrophysiological studies take advantage of transgenic mouse lines that specifically label SOM^+^ INs. The most commonly used transgenic GAD67-eGFP mouse lines labeling (sub)populations of SOM^+^ interneurons are the so-called GIN mouse line (GIN: GFP-expressing inhibitory interneurons [80]), the X98 and the X94 mouse line [57]. In all three mouse lines the random insertion of the GAD67-eGFP construct into the mouse genome resulted in a distinct eGFP labeling pattern in the cortex. Multivariate-analysis of the morphological and electrophysiological features of GIN, X98 and X94 SOM^+^ INs revealed that X98 and X94 SOM^+^ INs exhibit well-segregated, distinct types while GIN separated only partially from both types indicating a mixed subpopulation of SOM^+^ INs [57]. The authors concluded that the entity of SOM^+^ INs is composed of at least two different types of cells, with X98 cells mostly corresponding to LTS Martinotti cells and X94 cells representing quasi fast-spiking interneurons that morphologically resemble basket cells. Multivariate analyses of neocortical GIN alone later demonstrated that the population of GIN constitutes different subtypes of SOM^+^ INs ([56,62], somatosensory cortex; [12], cingulate cortex). It is therefore well-established that GIN themselves are composed of different subtypes of SOM^+^ INs; however, the number of potential types varies in all three studies (2–4 possible subtypes).

## 6. Synaptic Connectivity of SOM^+^ INs

### 6.1. Synaptic Input onto SOM^+^ INs

Whole-cell patch-clamp experiments from acute brain slices could show that supragranular SOM^+^ INs receive spontaneous synaptic inputs at a frequency of around 1 Hz [12]. However, only monophasic postsynaptic potentials were included in the analysis, therefore this number is likely higher. The excitatory input seems to come mainly from neighboring pyramidal neurons [98,99]. Given that L2/3 and L5 SOM^+^ INs have a relatively high input resistance and depolarized membrane potential, SOM^+^ INs are much more excitable compared to FS cells. Therefore, a single high-frequency burst elicited in the presynaptic pyramidal cell is able to trigger a spike in the postsynaptic SOM^+^ IN [59,100]. In addition, L4 and L5b SOM^+^ INs receive input from thalamic projections [101,102,103,104]. Intriguingly, layer 2/3/5 and layer 4 SOM^+^ INs receive highly complementary patterns of synaptic input, indicating that they are differentially recruited by activity in different cortical layers [98]. With the help of rabies virus injections it could be shown that barrel cortex SOM^+^ INs also receive corticocortical projections from the secondary somatosensory and primary motor cortex as well as from the contralateral somatosensory cortex and other brain regions [105]. Dual recordings in the visual cortex showed that stimulation of a single 2/3 pyramidal neuron can activate up to 30% of SOM^+^ INs within a 100 µm radius [100]. In contrast to synapses between pyramidal cells and fast-spiking interneurons, synapses between pyramidal neurons and SOM^+^ INs are generally facilitating exhibiting a nonlinear activity increase as the number of active pyramidal cells increases [97,104,106]. Moreover, with the help of paired recordings in acute slices it could be shown that SOM^+^ INs display a 66% probability of electrical coupling [107]. In addition, acetylcholine specifically depolarizes SOM^+^ INs (but not basket cells) pointing to a cholinergic innervation of SOM^+^ INs [97,103,108,109,110]. A cholinergic innervation of SOM^+^ INs could also be shown using rabies viral injections into the mouse barrel cortex and observing presynaptic projection neurons in the basal nucleus of Meynert [105]. In summary, SOM^+^ INs are not only excited locally by neighboring pyramidal cells, they are also activated by projection neurons from remote brain areas. In addition, SOM^+^ IN excitability is modulated by monoaminergic innervation. Serotonergic fibers originating from the nuclei raphé possibly represent another source of synaptic modulation of SOM^+^ INs [111]. While electrophysiological studies established a serotonergic modulation of FS and of 5-HT_3A_ receptor-expressing interneurons, serotonergic innervation of SOM^+^ INs has so far only been shown in electron microscopy studies and it remains to be tested whether serotonin modulates neocortical SOM^+^ INs on a global level or only in certain brain areas such as the visual cortex [112,113,114,115,116]. A dopaminergic modulation of prefrontal cortical interneurons via the D2 receptor was shown by Tseng and O’Donnell [117] in adult rats. Even though, D2-responsive interneurons were only classified as fast-spiking or non-fast-spiking, it is likely that SOM^+^ INs were part of the non-FS group. Interestingly, a D1-mediated effect on interneuron excitability was specifically shown for superficial VIP^+^ INs [118], indicating that cell type-specific innervation is a property of the postsynaptic cell and its receptors and not exclusively of presynaptic fiber specificity. Lastly, activation of alpha-adrenoceptors induces membrane depolarization and increased excitability in SOM^+^ INs whereas activation of beta-adrenoceptors weakens the synaptic transmission between the presynaptic SOM^+^ IN and the postsynaptic pyramidal neuron [119,120]. 

The inhibitory input onto SOM^+^ INs comes mainly from three sources: (1) VIP^+^ INs located in layer 1 [99,121,122,123,124], and (2) from basket cells [124,125,126]. The functional role of SOM^+^ IN inhibition by VIP^+^ or PV^+^ cells will be discussed in the following section.

### 6.2. Postsynaptic Targets of SOM^+^ INs

In layer 2/3 and in layers 5 and 6, pyramidal neurons represent the preferred postsynaptic target of SOM^+^ INs [59,75,76,127,128]. As can be seen from Figure 2, layer 2/3 and layer 5/6 SOM^+^ INs preferentially target pyramidal cell dendrites, whereas FS cells (PV^+^ INs), in comparison to SOM^+^ INs, exhibit a higher likelihood of targeting the pyramidal cell soma. The postsynaptic targeting bias of basket cell-like L4 SOM^+^ INs is currently not known but it is likely that they are also mostly dendrite-targeting interneurons [128]. Unlike Martinotti-(like) SOM^+^ INs, however, layer 5 non-Martinotti SOM^+^ INs do not target layer 2/3 or layer 5 pyramidal neurons, instead they seem to provide inhibition only to neurons in layer 4. 5-HT_3A_ receptor-expressing interneurons preferentially target pyramidal cell dendrites (in case of VIP^+^ 5-HT_3A_ receptor expressing interneurons [129]) or the perisomatic region of a pyramidal neuron (in case of CCK^+^ 5-HT_3A_ receptor expressing interneurons [130]). It should be noted however, that all inhibitory cells (not chandelier cells) innervate the dendritic shaft of pyramidal neurons and axo-somatic contacts between an inhibitory interneuron and a pyramidal cell constitute just a small fraction of the total synaptic output of inhibitory interneurons in the rat frontal cortex [131]. Axo-dendritic boutons in turn constitute 37–90% of total synaptic contacts of individual inhibitory non-pyramidal cells [131]. Nonetheless, it appears that distinct IN types exhibit postsynaptic target biases [59]. In addition, SOM^+^ INs inhibit other GABAergic cells; however, it is not known whether a similar postsynaptic target bias exists in postsynaptic GABAergic interneurons compared to pyramidal cells. Among the inhibitory interneurons whose activity is suppressed by SOM^+^ INs are VIP^+^ [122,132] and PV^+^ INs [123,133]. While superficial and infragranular SOM^+^ INs preferentially target pyramidal neurons, the main postsynaptic target of layer 4 SOM^+^ INs are FS interneurons that in turn target pyramidal cells [76]. It is currently not known whether granular SOM^+^ INs preferentially target the somatic vs. the dendritic region of a given pyramidal neuron. 

## 7. Role of SOM^+^ INs in Neuronal Circuits

### 7.1. Feedback Inhibition/Lateral Inhibition

The best studied role of SOM^+^ INs within a neocortical circuit is that of providing feedback inhibition and/or lateral inhibition to the same and/or neighboring pyramidal neurons [102,103,106]. In doing so, layer 2/3 and layer 5 SOM^+^ INs receive excitatory input from neighboring pyramidal cells and in turn, either provide feedback inhibition onto the same pyramidal neuron or inhibit neighboring pyramidal neurons to increase the contrast of competing inputs [103,134,135]. In line with this idea, SOM^+^ INs were shown to contribute to surround suppression in the visual cortex V1 [134,136] and to frequency tuning in the auditory cortex [137]. Given the fact that synapses between a presynaptic pyramidal neuron and a postsynaptic SOM^+^ IN are strongly facilitating, feedback inhibition onto the same or neighboring pyramidal cell is dependent on the firing frequency in the presynaptic pyramidal neuron, i.e., SOM^+^ INs act as rate detectors providing a frequency filter to the postsynaptic pyramidal neuron.

### 7.2. Feed-Forward Inhibition

Layer 2/3 and layer 5 SOM^+^ INs not only receive excitatory input from neighboring pyramidal neurons but also from corticocortical and thalamic afferents, therefore, it is assumed that they are also involved in providing feed-forward inhibition to pyramidal neurons [138]. Likewise, layer 4 SOM^+^ INs of the barrel cortex receive monosynaptic excitatory thalamic input [104]. However, unlike layer 2/3 and layer 5 SOM^+^ INs, layer 4 SOM^+^ INs target FS interneurons with a higher probability compared to pyramidal neurons.

### 7.3. Disinhibition

**Figure 3:**
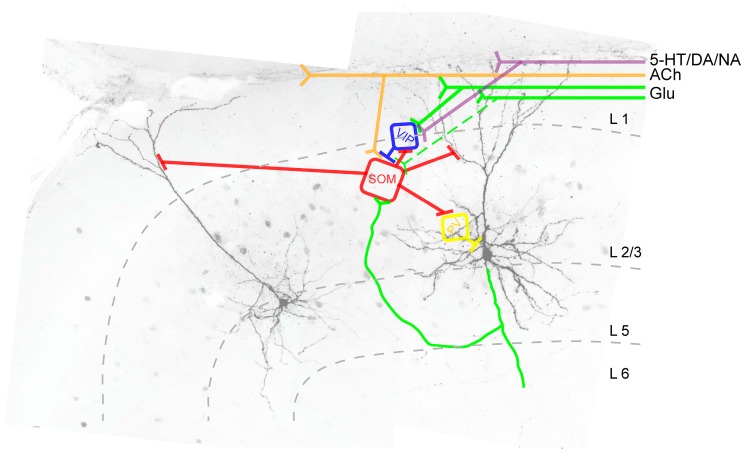
Intracortical synaptic connections between SOM^+^ INs (red) and pyramidal neurons (black) and between SOM^+^ INs and other GABAergic interneurons (blue, yellow) in L2/3 of the agranular frontal cortex. Abbreviations: SOM: SOM^+^ IN; VIP: Vasoactive-intestinal peptide-expressing interneuron; PV: PV^+^ IN; Glu: glutamatergic fibers; ACh: cholinergic fibers; 5-HT: serotoninergic fibers; DA: dopaminergic fibers; NA: noradrenergic fibers, L: cortical layer.

Layer 1 VIP^+^ INs preferentially target SOM^+^ INs (see Figure 3), thereby providing pyramidal cell disinhibition [122,139]. In order to answer the question of a possible functional role of this disinhibitory circuit, several recent studies combined in vitro or in vivo electrophysiological recordings with optogenetic manipulation of SOM^+^ INs and animal behavior ([99,139,140,141,142] (barrel cortex) and [143,144,145] (visual cortex)). However, due to different technical approaches and partially contrasting findings, a direct comparison of these studies is difficult. The role of VIP^+^ IN-induced pyramidal cell disinhibition during whisking can be summarized as follows: active whisking activates VIP^+^ INs and differentially modulates the activity in subsets of SOM^+^ INs in the barrel cortex. It is suggested that VIP^+^ INs are recruited by the motor cortex and in turn, inhibit SOM^+^ INs to allow for a more permissive pyramidal cell dendrite. Analogous to the somatosensory cortex, active movement also modulates SOM^+^ IN activity in other primary sensory brain areas, such as the visual cortex leading to reduced surround suppression [146]. 

Unlike layer 2/3 and layer 5 SOM^+^ INs, layer 4 SOM^+^ INs provide disinhibition of pyramidal neurons by specifically synapsing onto PV^+^ INs that in turn project onto pyramidal neurons [76,123,147]. As a consequence of this SOM^+^ IN-induced disinhibitory circuit, optogenetic silencing of SOM^+^ INs increased the firing rates of superficial pyramidal neurons whereas those of L4 pyramidal neurons were decreased. It is suggested that this disinhibitory circuit tunes the output of layer 4 pyramidal neurons, and as such, that this disinhibition could play an important role for cortical information processing [76].

## 8. Role of SOM^+^ Interneurons in Sensory Processing and in Learning and Memory

Based on what is known about SOM^+^ INs, this section tries to derive a functional role of SOM^+^ INs in the living animal.

Any specific sensory information reaches its associated primary sensory brain area via specific first-order thalamic relay nuclei. For example, primary sensory information of whisker touch reaches the barrel cortex via specific nuclei of the somatosensory thalamus (ventral posterior medial nucleus; [148]). Most of these excitatory thalamic afferents target the dendritic shaft of pyramidal cells in layer 4 (bottom-up inputs; [149]). In addition, secondary sensory information, i.e., the integrated signal of whisker touch from associated brain areas or from higher-order thalamic relay nuclei mainly innervates the distal apical dendrites of pyramidal neurons in layer 1 and avoids layer 4 (top-down inputs, [150]). Thus, the same postsynaptic pyramidal cell is differentially innervated by two types of stimuli, a specific short-latency sensory stimulus and its integrated longer-latency, second-order stimulus. Given that SOM^+^ INs preferentially target the apical dendrites of pyramidal cells, it is believed that their activity paves the way for either bottom-up or top-down inputs [151,152]. Habituation is defined as a loss of central response during continued or chronic exposure of a given stimulus. With the help of calcium imaging and unit recordings in the auditory cortex of awake, head-fixed mice that had been continuously exposed to a specific sound, it could be shown that this reduction in excitatory responses of layer 2/3 pyramidal neurons was accompanied by increased activity of SOM^+^ INs and reduced activity in PV^+^ cells [153]. A reversal of this habituation by combining the habituated sound to a behavioral task increased layer 2/3 pyramidal cell responses by specifically reducing SOM^+^ IN activity, indicating that SOM^+^ INs are involved in a bidirectional modulation of sensory inputs. Similar to habituation, Natan and colleagues [154] reported that SOM^+^ INs play a role in short-term stimulus-specific adaptation in mice that had been presented a trained, standard and a deviant tone. It could be shown that the SOM-mediated inhibitory drive increased with repeated presentations of the standard tone. The fact that coupling a sensory stimulus (e.g., a tone or light) to a behavioral task affects habituation by modulating the activity of SOM^+^ INs can be explained by a differential recruitment of SOM^+^ INs as a function of the behavioral task. For example, spatial integration and surround suppression in V1 in mice is altered by locomotion, which can be explained by the fact that locomotion activates VIP^+^ INs leading to reduced activity in SOM^+^ INs [144,146]. Therefore, it seems that the activity of SOM^+^ INs has a direct, context-dependent, impact on the responsiveness of pyramidal neurons towards a sensory stimulus.

Moreover, SOM^+^ INs seem to play a role in memory and learning and their activity is modulated in those brain areas that are associated with a learning or memory task [155]. For example, classical fear conditioning of a whisker stimulus increases the density of SOM immunoreactive cells in layer 4 of the stimulus-specific barrel cortex area that is accompanied by an increased density of inhibitory synapses. Similarly to classical fear conditioning, a motor learning task results in a specific decrease in the number of presynaptic axon boutons from SOM^+^ but not from PV^+^ interneurons in layer 1 of the motor cortex [156]. Loss of presynaptic axon boutons was accompanied by a reduction in the spine density of the distal dendrites of layer 2/3 pyramidal neurons. Optogenetic enhancement or suppression of SOM^+^ IN activity in turn destabilized respectively hyper-stabilized spines. Moreover, a role of SOM^+^ INs in maintaining the content of working memory during the delay period of a working memory task was suggested by Kim and colleagues [157]. They could show that SOM^+^ IN firing in the medial prefrontal cortex was increased during the delay period of mice performing a spatial working memory task and that optogenetic stimulation of SOM^+^ INs during the behavioral task impaired the animal’s performance in the working memory task. Impairment of memory-guided behavior after activation of SOM^+^ INs was also shown by Kamigaki and Dan [158].

The fact that many SOM^+^ INs express dendritic spines themselves adds another layer of complexity to our current knowledge on SOM^+^ IN function in learning and memory. It is known that dendritic spines on inhibitory interneurons can undergo structural changes [159] and that they are regulated in an experience-dependent manner. Sensory deprivation of interneurons in V1 results in a rapid loss of dendritic spines and axonal boutons from inhibitory interneurons [160], many of which are NPY^+^. Given that NPY and SOM are often coexpressed in the same interneuron, SOM^+^ interneuron dendrites might also be affected by sensory deprivation.

## 9. Role of SOM^+^ Interneurons in Mood Disorders

The most common mood disorders comprise major depressive disorder, substance-induced mood disorder and bipolar disorder. It is estimated that around 20% of people (in North America) have undergone a depressive episode at any one time. In spite of its high prevalence, the underlying mechanisms that lead to major depressive disorder are unclear, possibly owing to its wide variety of clinical symptoms ranging from anhedonia and sleep disorders to weight gain or loss [161]. One brain region that is believed to play a role in mood disorders is the prefrontal cortex; as such, I will briefly summarize intersections between SOM^+^ INs, mood disorders and the prefrontal cortex. As mentioned, the prefrontal cortex plays a key role in working memory, and as such, must integrate behaviorally-relevant information from different brain areas (such as sensory, motor or limbic brain areas for example) to respond with an adequate behavioral performance [162]. The subgenual cingulate cortex is part of the prefrontal cortex, and its physiological activity seems to be affected in mood disorders [163]. Growing evidence suggest that SOM^+^ INs of the prefrontal cortex are especially affected by major depressive disorder and postmortem analyses of human brains revealed reduced somatostatin levels [6,7]. It is currently unknown why SOM^+^ INs are especially vulnerable to major depressive disorder; one hypothesis is that these neurons lack trophic brain derived neurotrophic factor (BDNF) support [164,165,166]. Disinhibition of SOM^+^ INs induces a sustained anti-depressant-like phenotype that includes behavioral and biochemical endpoints of anti-depressant drug treatment [167,168]. On the other hand, intracerebroventricular infusion of the somatostatin peptide into rat brains resulted in anxiolytic and antidepressant-like behavior [169]. 

However, it is noteworthy that while the neuropeptide SOM can affect neuronal excitability in multiple ways [170,171,172,173], it is currently not known under which conditions (and if at all) SOM is in fact released by SOM^+^ INs [174]. In addition, it is currently not known whether and in what way a reduction of SOM levels in SOM^+^ INs affects their function. Studies showing that major depressive disorder has a functional impact on SOM^+^ IN physiology are missing [175], even though a correlation between SOM, SOM^+^ INs and major depressive disorder cannot be denied. 

## 10. Outlook and Future Avenues in SOM^+^ IN Research

Our understanding of the functions and connectivity of SOM^+^ INs has greatly increased over the past decade; however, it is currently not known whether different subtypes of SOM^+^ INs within a given cortical layer have a similar functional impact on postsynaptic pyramidal neurons. In vivo manipulations of SOM^+^ INs activity point to a differential activation of SOM^+^ IN subtypes. By extending analyses of the cortical connectivity matrix, the specific roles of SOM^+^ INs within the neuronal circuitry of the neocortex will hopefully be further elucidated and their function further specified.

## Abbreviations

5-HT	5-hydroxytryptamine
ACh	Acetylcholine
AHP	Afterhyperpolarization
Ascl-1	Achaete-scute homolog 1
BDNF	Brain-derived neurotrophic factor
CCK	cholecystokinin
CGE	Caudal ganglionic eminence
CoupTF2	Coup transcription factor 2
D1	Dopamine receptor 1
D2	Dopamine receptor 2
DA	Dopamine
Dlx-1/-2	Distal-less homeobox ½
FS	Fast-spiking
GABA	Gamma aminobutyric acid
GFP	Green fluorescent protein
GIN	GFP-expressing inhibitory interneurons
L	Cortical layer
LTS	Low-threshold spike
MGE	Medial ganglionic eminence
NA	Noradrenaline
nNOS	Neuronal nitric oxide synthase
NO	Nitric oxide
NPY	Neuropeptide Y
POA	Preoptic area
PV	Parvalbumin
RSNP	Regular-spiking non-pyramidal
SOM	Somatostatin
V1	Primary visual cortex
VIP	Vasoactive intestinal peptide
